# Changing course: supporting a shift to environmental strategies in a state prevention system

**DOI:** 10.1186/s13011-020-00341-y

**Published:** 2021-01-12

**Authors:** Parissa J. Ballard, Melinda Pankratz, Kimberly G. Wagoner, Jennifer Cornacchione Ross, Scott D. Rhodes, Sunday Azagba, Eunyoung Y. Song, Mark Wolfson

**Affiliations:** 1 Wake Forest School of Medicine, Family & Community Medicine, Piedmont Plaza Building 1, 1920 W 1st St., Winston-Salem, NC 27104 USA; 2 Wake Forest School of Medicine, Social Sciences and Health Policy, Piedmont Plaza Building 1, 1920 W 1st St., Winston-Salem, NC 27104 USA; 3 University of Utah School of Medicine, Salt Lake City, USA; 4 Health Quality Partners, Doylestown, PA USA; 5 University of California, Riverside, USA

**Keywords:** Substance misuse prevention, Environmental strategies, Systems change, State prevention system, Policy

## Abstract

**Background:**

This study examines how the North Carolina state prevention system responded to a policy shift from individual-level prevention strategies to environmental strategies from the perspective of the organizations implementing the policy shift.

**Methods:**

We use two data sources. First, we conducted interviews to collect qualitative data from key informants. Second, we used prevention provider agency expenditure data from the year the shift was announced and the following year.

**Results:**

The interviews allowed us to identify effective features of policy change implementation in complex systems, such as the need for clear communication and guidance about the policy changes. Our interview and expenditure analyses also underscore variation in the level of guidance and oversight provided by implementing agencies to prevention providers.

**Conclusions:**

Our analyses suggest that more active monitoring and oversight may have facilitated more consistent implementation of the policy shift toward greater use of environmental prevention strategies.

## Background

Substance misuse is a major public health issue in the United States. The toll on health and human lives and the economic burdens of substance misuse have been extensively documented [1–4]. It is generally believed that effectively addressing the problem of substance misuse at a population level requires widespread delivery of a continuum of services, ranging from prevention and early intervention to treatment and recovery support [5, 6].

### National Prevention Efforts: substance abuse prevention and treatment block Grant (SABG)

Prevention services in the United States are primarily supported by the Substance Abuse Prevention and Treatment Block Grant (SABG), mandated by law to provide funding and technical assistance for substance misuse services [7]. The Substance Abuse and Mental Health Services Administration (SAMHSA) awards a block grant to each of the 50 states, the District of Columbia, Puerto Rico, the U.S. Virgin Islands, six Pacific territories, and one tribal entity. The state agencies responsible for overseeing the SABG, customarily referred to as the “Single State Agency” (SSA), are charged with providing treatment to individuals without insurance; funding evidence-based treatment and support services that are not covered by Medicaid, Medicare, or private insurance; and funding primary prevention planning and implementation [7]. The SABG mandates that 20% of each award be used for primary prevention services [8]; this meets an essential need as one of the few mechanisms that fund prevention. Other funding sources include SAMHSA’s discretionary grants such as the Partnerships For Success grants [9] and the Strategic Prevention Framework (SPF) – Rx [10], and the Centers for Disease Prevention and Control’s Drug-Free Communities [11].

SSAs have flexibility in how the SABG program is administered, resulting in different program implementation structures by state [7, 12, 13]. For example, some states have “super-agencies” with authority to make decisions about prevention policy and funding allocations, while others have coordinating bodies to integrate prevention efforts, and some have neither [12]. However, there is a dearth of evidence about how different state prevention systems operate to support prevention providers [14]. Moreover, there is little research on how state prevention systems respond to shifts in the evidence and understanding of effective prevention strategies, such as the growing appreciation of potential population-level impact of policies and other environmental approaches to prevention. This gap in the science of how states respond to prevention innovations (e.g., environmental strategies) stands in marked contrast to research on state substance abuse treatment systems, where there has been extensive research on performance measurement, system reach and effectiveness, and shifts in treatment modalities and payment mechanisms [15–17].

### Shifting prevention landscape: a focus on environmental strategies in substance misuse prevention

Efforts to prevent substance misuse have typically taken one of two broad approaches [18]. The first approach focuses on changing *individuals*, through interventions intended to change attitudes and intentions, provide knowledge, and develop skills to help individuals resist influences that would otherwise lead them to misuse substances [18]. Individual-level prevention strategies often involve repeatedly working with individuals and small groups, which may require heavy resource investment to reach a significant portion of a target population (e.g., a city or town, county, or state [19, 20],).

In the second approach, the focus of intervention shifts from changing individual behaviors to changing the *environments* that shape these behaviors [20–23]. The core insight of these approaches is that trying to change the “hearts and minds” of individuals, without paying commensurate attention to factors in the larger environment that support unhealthy behaviors, will have a limited impact, at best. Such strategies are recognized in public health research as having high potential to effectively promote long-term health at the population level by reaching broad segments of society and requiring less individual effort [19, 20]. With regards to substance misuse, research has shown that modifying policies, practices, and social norms can significantly reduce rates of use and problems associated with alcohol and tobacco use [24–29].

SAMHSA recommends environmental approaches as one of six Center for Substance Abuse Prevention (CSAP) strategies for primary prevention in its Strategic Prevention Framework (SPF). The six strategies focus on “establish [ing] or chang [ing] written and unwritten community standards, codes, and attitudes...to influence the general population’s use of alcohol and other drugs” [5]. SAMHSA defines environmental strategies as those “aimed at the settings and conditions in which people live, work, and socialize” [30]. SAMSHA’s guidance is informed by the socio-ecological model and considers the multiple contexts that shape substance use behavior [30]. Within the socio-ecological framework [31] environmental strategies can be considered as potentially targeting broader ecological contexts of substance use—norms, policies, laws, and culture—as opposed to more immediate contexts such as families and classrooms. Examples of environmental strategies include policies that increase product price (e.g., tobacco, alcohol), limit the prescribing of opioid analgesics, enact minimum legal age for the use of substances such as alcohol and tobacco, and related enforcement efforts [32]. Substance use is a complex problem that is influenced by factors at multiple levels. Therefore, comprehensive approaches that include both environmental and individual-level strategies are needed.

### Implementing a state-level prevention policy shift

State-level prevention policies shape local and day-to-day prevention efforts [13, 14]. However, there is a dearth of research examining state-level substance misuse prevention systems. This is a critical gap because documenting how state-level policies are enacted within complex state prevention systems will build knowledge and apply best practices across prevention system models. Research on policy implementation, including evidence from implementation science, suggests that implementing policy changes in complex systems requires multi-level efforts and attention to local contexts [33–35]. In complex state health and substance misuse prevention systems [7, 36], successful system change is thought to require understanding, support, and endorsement of new policies on the part of regional and local implementing agencies as they are the ones putting policies and programs into practice in communities (e.g. [37, 38]. This paper harnesses concepts from implementation science to understand changes in prevention policy in complex state substance misuse prevention systems.

### North Carolina’s prevention system: regional entities

The Department of Mental Health, Developmental Disabilities, and Substance Abuse Services (hereafter referred to as NC DMH) is the SSA responsible for administering the North Carolina Substance Abuse Prevention and Treatment Block Grant (hereafter referred to as “the block grant”). The block grant provides approximately $45 million per year for substance misuse prevention, early intervention, treatment, and recovery support, about $9 million of which is specifically allocated to substance misuse prevention [5]. The agencies that implement the block grant in North Carolina are regional Local Management Entities/Managed Care Organizations (LME-MCOs). There are seven LMC-MCOs in NC, with catchment areas ranging from 4 to 26 counties [39]. LME-MCOs contract with local prevention agencies, referred to hereafter as “prevention providers,” who are the organizations delivering prevention programming across the state.

Although each organization is different, LME-MCOs are usually non-profit agencies that work closely with the state as contractors for grant fulfillment. LME-MCOs implement state-level policies, and a typical array of services includes: recruiting and maintaining prevention provider networks, administering block grant funding, and providing oversight and guidance to their contracted prevention providers [40]. The LME-MCOs have discretion in how these goals are achieved. For example, each LME-MCO decides whether and how to review strategies being implemented by prevention providers and defines whether and what contractual requirements to implement beyond those required by NC DMH.

### North Carolina prevention system shift to environmental strategies: new benchmarks for prevention

Aligning with the national trend toward increased use of environmental strategies for substance misuse prevention [41], in September of 2016, the NC DMH issued new “Benchmarks for Prevention” (hereinafter referred to as “the benchmarks”, [42]. The benchmarks’ goal was to guide LME-MCOs to work towards using more environmental strategies through the prevention block grant funds [42]. The benchmarks did so by specifying budget amounts to be spent on different prevention strategies. The benchmarks set a target of LME-MCOs expending at least 51% of their allocated block grant funds on community-based processes and environmental strategies and no more than 30% of their block grant funds on prevention education, 12% on information dissemination, 4% on problem identification and referral, and 3% on alternative activities. The new benchmarks were communicated to LME-MCOs with a flexible timeline, instructions for how to meet the benchmarks, and guidance to support the transition. For example, LME-MCOs were given a model scope of work that the LME-MCOs could use in contracting with prevention providers [40].

**Fig. 1:**
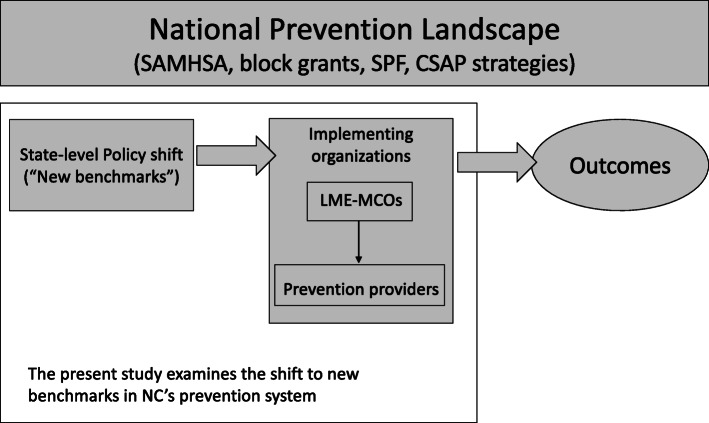
Prevention policy to practice: A Conceptual framework for understanding a policy shift in North Carolina’s prevention system

### The present study

In this study, we sought to better understand the state-level shift to emphasizing environmental strategies in prevention policy, from the perspective of regional entities directly involved in managing the shift in North Carolina (Fig. 1). We answer the following research questions: (1) What were LME-MCO staff perceptions of the shift to new benchmarks (i.e., increased use of environmental strategies)? (2) What strategies did LME-MCOs use to transition their network to meet the benchmarks? (3) How successful were the LME-MCOs in meeting the benchmarks? (4) Were strategies employed by LME-MCOs to meet the benchmarks related to their success in actually doing so?

## Methods

Our research team at the Wake Forest School of Medicine was commissioned to evaluate the prevention component of the North Carolina block grant by the NC DMH [40, 43, 44]. This paper draws on both qualitative and quantitative data collected during the first phase of the evaluation. Mixed methods studies are especially useful in the context of translational and systems-oriented projects because the two types of data provide complementary perspectives regarding complex system-level changes [45]. We present first-person accounts about state-level changes in prevention from key stakeholders (i.e., prevention representatives from LME-MCOs, Part 1) and overlay qualitative findings with quantitative data describing how successfully the new benchmarks were met (Part 2).

### Participants and procedures

We used data from two sources to answer our research questions: qualitative data from key informant interviews and quantitative expenditure data from SAMHSA annual reports from fiscal year (FY) 2017 and 2018.

### Part 1. Key informant interview procedures

We used a purposive sampling strategy, specifically a key informant technique [46], to obtain qualitative data from one or more representatives from each LME-MCO. We interviewed the individual or individuals at each LME-MCO who were most knowledgeable about the organization’s work in substance use prevention. We sent an invitation to each LME-MCO SABG Contract Manager (*N* = 7), requesting permission to interview their prevention point of contact and identifying other individuals who were knowledgeable about the prevention component of the SABG. We conducted interviews as one-on-one conversations with three LME-MCOs and group interviews with four LME-MCO representatives with three other LME-MCOs between February and March 2019. One LME-MCO chose not to participate in the interviews. In total, we conducted six interviews with 15 people, who represented six out of the seven LME-MCOs in North Carolina.

Those who agreed to participate were contacted via email to schedule a 60-min interview, to be conducted either in-person or by phone. The email described the purpose of the LME-MCO interviews to gain their perspective as key stakeholders in the block grant program. Semi-structured interviews were conducted by four members of the research staff. Interviews asked about the participants’ perceptions of the shift to new benchmarks, their interactions with providers in their network, and their general views about substance misuse prevention. Two members of the research staff were present at each interview. Five were conducted in person, and one was conducted by phone. All interviews were recorded and professionally transcribed.

### Part 2. SAMHSA annual reports in fiscal years 2017 and 2018

The quantitative data used in this analysis come from the North Carolina Division of Mental Health’s Block Grant Compliance Reports from fiscal year (FY) 2017 and 2018. The reports are submitted by LME-MCOs and contain data on expenditures and people served by CSAP strategy.

### Analysis

#### Part 1

Interview data were coded using a thematic analysis approach [47, 48]. We used a realist theoretical framework, treating interviews as direct reflections of participants’ experiences [48]. Aligned with thematic approaches, we did not use a codebook or calculate inter-coder reliability; our analysis was both deductive (themes were informed by our specific topics of interest) and inductive (themes were informed by the data [48]). Two analysts (first and second authors) independently familiarized themselves with the data, generated initial codes, searched for themes, reviewed themes, defined and named themes, and generated a summary report collaboratively and with feedback from the full evaluation team. The final phases (defining themes and generating a report) were focused on the themes that arose from the portion of the interview relevant to the change to new benchmarks, rather than for all themes we identified in our data. We then organized the themes into three key findings.

#### Part 2

To understand how effective LME-MCOs were in meeting the new benchmarks, we conducted a descriptive analysis of funding expenditures data. These data were compiled from reports by NC prevention providers outlining their funding expenditures by CSAP strategy to their LME-MCOs (i.e., NC Substance Abuse Prevention and Treatment Block Grant Compliance Reports). These data are in the form of dollar amounts spent in each CSAP strategy in FY 2017 (when the benchmarks were announced) and 2018 (when LME-MCOs first started transitioning to the benchmarks). We calculated the percent of funding spent on each CSAP strategy by LME-MCO.

In addition, to further explore the role of LME-MCO to support and provide oversight to their providers, we conducted an additional analysis examining funding expenditures by strategy across groups coded as *intensive* and *facilitative* oversight. We stratified LME-MCOs into two groups based on the level of oversight and guidance these organizations supplied to the providers in their network: an *intensive oversight* group and a *facilitative oversight* group. The *intensive oversight* group consisted of LME-MCOs that took proactive actions to encourage compliance with the benchmarks and to ensure their providers were performing well. This group used strategies such as issuing a request for proposals to narrow the scope of prevention providers to those best able to meet the new benchmarks; using the model scope of work developed by the NC DMH to provide accountability for meeting the benchmarks, and developing an in-depth provider audit system to increase accountability. In general, LME-MCOs in this group had more frequent or extensive or varied in purpose contact with their providers. LME-MCOs in the *facilitative oversight* group used only oversight strategies that were required of them, such as semi-annual compliance reports in Excel and monthly reports in Ecco (a data collection and management system). Their contact with providers was less frequent and extensive, and more collaborative.

## Results

### Part 1

We identified ten themes from the interviews that we then organized into three key findings: 1) the shift to new benchmarks was viewed as a positive step, overall (with some challenges highlighted); 2) the LME-MCOs changed many of their practices as a result of the shift to new benchmarks; 3) there were mixed perceptions about the role of LME-MCOs in the system. Table 1 summarizes the key findings and themes.

**Table 1:** Summary of key findings and themes

Key findings	Theme
1) Benchmark rollout perceived positively	1) Instructions and guidance about new benchmarks were clear and helpful
	2) Timeline to achieve new benchmarks was sufficient
	3) Challenge identified: staff capacity and expertise
	4) Challenge identified: changes to prior program investments
2) LME-MCOs made tangible changes to meet benchmarks	5) Increased monitoring and oversight of providers
	6) Variation in guidance/oversight strategies (Intensive strategies and facilitative strategies)
	7) Minimal changes in selecting providers and amounts of funding
3) Mixed perceptions of the role of LME-MCOs within the state prevention system	8) Prevention as a small part of work within the scope of work LME-MCOs do
	9) Impressions that managed care system is complex
	10) Perceived limitations of changes that LME-MCOs can make

#### Key finding 1: benchmark rollout perceived positively

The benchmarks’ goal was to guide LME-MCOs to work towards using more environmental strategies in prevention across the state of NC. This confronted LME-MCOs with a major change. Though challenges were identified, overall, participants from LME-MCOs reported positive perceptions of how the new benchmarks were rolled out. For example, participants expressed a strong sense that the benchmarks and guidelines given were clear. Participants also said that they felt equipped to meet the policy shift’s challenges because the instructions were clear (Theme 1). For example, one interviewee said:

*… It was a September 2016 memo that was the clearest instruction we have received. I’ve been involved in prevention since the early 2000s, and it was the clearest thing I’ve ever seen in terms of how they wanted funding to be distributed. We pretty much used that as the basis to inform our RFP process... They had given direction about the three CORE strategies, and so we included that in our process...*

Others also expressed that the timeline for meeting the new benchmarks was achievable (Theme 2). Participants discussed how they received warning that a policy shift would be coming well in advance of implementing changes and how they had adequate time to make changes, as opposed to unrealistic expectations for a rapid shift to the new funding allocations of the benchmarks. For example, one participant explained:

*... We were prepared all along...change is coming. If it didn’t come, it’s soon to come. With that memo, we were given the opportunity to ask questions before it [was] shared with our prevention network. I remember, there were calls. There [were] monthly point-of-contact calls. We talked about upcoming changes.*

Although the overall shift to new benchmarks was experienced positively, participants also mentioned encountering challenges. Some participants noted that the shift to new benchmarks was a major change, which can be challenging and come with frustrations. Specific challenges had to do with LME-MCO programmatic capacity and preferences (Theme 3). For example, some LME-MCO staff felt that they lacked personnel with expertise in environmental strategies and could not change and adapt quickly to support these new strategies. In addition to staff capacity, some noted that demands of transition—which required staff with the skills to investigate environmental strategies and to form new relationships with prevention providers who were implementing such strategies—were large relative to staffing resources available (Theme 4). Others mentioned that the new benchmarks were difficult because they liked school-based strategies and felt invested in existing programs as well as existing relationships with providers. For example, one interviewee explained: *“I think there is some disappointment because... [prevention providers] used to be able to do more stuff within the school system that they can’t do now, had to go straight to community-based things. I think that’s been disappointing for folks.”* In sum, while making the shift to environmental strategies was identified as challenging and came with frustrations, participants identified specific aspects of the way the shift was handled that made it seem reasonable and achievable, namely, clear instructions and appropriate time and support were provided by the state.

#### Key finding 2: LME-MCOs made tangible changes to meet benchmarks

A second key finding was that the six LME-MCOs made several important changes in their practices as a result of the shift to new benchmarks. We specifically asked how the LME-MCOs structured their funding, oversight, and guidance to the providers in their network. Almost all of the interviewees said they increased LME-MCO monitoring and oversight of providers in their network following the shift to new benchmarks (Theme 5). Importantly, we identified variation in how this guidance and oversight was provided (Theme 6). Some LME-MCOs implemented an intensive oversight process to ensure that their providers met the requirements for prevention strategies in response to the shift to new benchmarks. Examples of intensive oversight processes include rebidding their network (i.e., putting out a request for proposals that required providers to re-apply for funds). Another example is adding requirements beyond those required by the state, such as creating mandates for providers to demonstrate coverage of their entire service areas and creating more detailed forms and audit tools compared to the forms required by the state. Some participants mentioned trying to draw on data sources (such as Ecco data and Substance Abuse Prevention and Treatment Block Grant reporting) to support providers and encourage providers to make data-driven decisions. In one example of an intensive oversight process, one participant said:

*Right now, we have touch-point calls with our providers at least quarterly, and some of the ones who are struggling, much more often than that. We also are trying to look at the Ecco data, and when we cannot find what we need in Ecco, we are reaching out to providers directly to get it. We do desk reviews of their documentation periodically. A lot of it is talking and listening to what they are trying to do. We rely a great deal on the biannual [block grant] reporting, because it is the most concrete sort of numerical data that we can get at this point. I mean, we try to do our own, but that is where we see spread out all in one place the expenditures and the efforts that the providers are making. We follow up on those with the individual providers when we see issues.*

Other LME-MCOs relied on more supportive and facilitative oversight processes. For example, having regular meetings with providers to make collaborative decisions about prevention strategies, connecting providers to resources, and participating in provider-led events. As one participant described, *“We...try to participate in their events or what have you that they have going on that we feel like we can engage in that would be supportive. Because it opens up that line of communication. It makes it easier*.” Another example of facilitative oversight is that some LME-MCOs made sure providers knew that they were available and willing to support providers as-needed. “*Prevention providers have the opportunity to invite me, or any other staff, to their meetings. Or if they have questions, they send those questions up to me before they meet, to ensure that all the [counties in their service area] are on the same wavelength”*.

In contrast, many participants described an area where they did not make changes: in the selection of providers to fund and the level at which they were funded (Theme 7). Overwhelmingly, participants described being constrained in adding new providers to their network and, in some cases, limited in their ability to drop providers from their network. Some participants cited their perceptions that there could be political repercussions to dropping existing providers, even though there was no formal policy prohibiting them from making changes to which providers are funded. One participant noted:

*We don’t really have the latitude to sanction or any of that in terms of financial penalties or any of those sort of things … Some of that limiting comes more from a political arena. We started going down the road in the past. Now, I’m not talking about recent past as much … It appears that the state has its preferred providers and that there is some political connection related to that.*

Thus, taken together, we found that LME-MCOs made changes to meet the new benchmarks, especially in oversight and guidance, using a variety of strategies for this oversight and guidance. There was less change when it came to deciding which providers to fund.

#### Key finding 3: the role of LME-MCO within the state prevention system

While we did not explicitly ask LME-MCO participants about the way prevention is structured in NC, many shared their views on the topic. We identified three main ideas: the small role of prevention within the LME-MCOs’ scope of the work (Theme 8); their impressions of the overall managed care system (Theme 9), and perceived limitations of changes that LME-MCOs could make (Theme 10).

Several participants mentioned that prevention is a relatively small proportion of their overall budget, with the greatest share of their budget earmarked for substance abuse treatment. Consequently, some LME-MCOs staff expressed insufficient staff expertise and capacity to devote to the prevention portion of their work. For example, one participant described how she was assigned to her role in prevention as a new staff with no prevention training. Another interviewee explicitly mentioned that LME-MCOs do not receive funding for managing the prevention portion of the block grant.

Relatedly, participants overwhelmingly described their role in prevention as contract administrators. For some, this was evident in how they described prevention providers as experts. One participant explained:

*To get back to how they select their prevention model that they're going to use, they are really good about … they are the professionals, those folks working in the communities, they're the prevention professionals. They really look at what their communities need, and they make that decision what's going to work best for their communities and schools and the populations that they serve. We're more along for guidance, but they are really great at this. All of the providers that we work with have been doing this for a very long time.*

For others, this was evident in how they described their role as mostly connecting providers with resources, such as the Training and Technical Assistance (TTA) Center. Several participants expressed that they did not have expertise in prevention, but they could rely upon guidance and resources provided by the state, including the TTA Center, guidance documents, and regional meetings with LME-MCOs and provider agencies. As one interviewee stated, *“We’re going based on what the recommendation is from the state.”* Another elaborated on connecting providers with the TTA Center, explaining that:

*I feel like the training center has been extremely helpful in informing the providers on some successful strategies and helping them develop plans. I mean, during these trainings I actually sit with them individually and talk about what their plans are and how to implement it and everything. I think that that’s been extremely helpful.*

In addition to sharing thoughts on their own role within the overall managed care system in NC, there was some sense that the system is overly complicated. For example, one participant explained,

*When I tell people [about the] system of care and I'm managing block grants and prevention, they're like, "What?" It's like it doesn't make sense. The community connection piece of it is what makes sense to us because we're very connected with our providers, and very connected with our community stakeholders. We know where they identify needs. That's how, I think, we got put in the mix, but it is definitely a moving target.*

A third idea that arose was a sentiment that LME-MCOs are limited in what they can do. This came up in the context of the “needs and gaps analysis” that LME-MCOs are required to complete annually. Some participants expressed their perception that the needs and gaps analysis was informative but didn’t necessarily lead to change because they could not use the information given state-level funding restrictions. For example, when asked about the extent to which the needs-and-gaps analysis inform how they allocate funds to prevention providers, one interviewee responded: *“The block grant funds come down the state—come from the state with a lot of influence from the state, so it doesn’t inform us.”* Another stated:

*In a lot of ways, it really restricts us as to what we might want to do, but—because of the limited amount of funding and because those are locked in in such a way that it’s—we may make reference to some of those things within the gap-analysis piece, but it’s clearly not—I think the block grant— the funding really drives what our capability is and what those dollars are.*

Another interviewer shared this sentiment and gave a specific example.

*It currently does not in the arena of prevention. I’ll just give one example. I would definitely see in this space where we’re talking about substance abuse in children or teens where there’s probably good indications that prevention could have been something we could have targeted if we felt we had that latitude. We don’t. Several years running...we continued to have a gap indicator that we were really missing youth substance use for 13 to 26-year-olds, their age range. ...Addressing that early enough. Then when it crossed over into more of a serious issue and/or abuse or use pattern that could lend itself towards disorder by the time they got into their mid to late-20s, we were missing that … .all of a sudden, we had disordered people in their mid-20s and older that had never tracked earlier...Our gap in needs analysis pointed this out for several years. ...prevention was certainly one arena, that we had less impact on—partly because there’s not enough funding to really do that work in the adequate way it would need to be done…We have to go where the money is for solutions. It’s not in prevention.*

Participants also shared their views on the managed care system and their role within it. They felt they had a facilitative role, in some cases, they perceived that their power to implement changes was constrained. Overall, they expressed the sense that they deferred to the prevention providers as the true prevention experts, and some expressed that they felt the need to provide more intensive oversight.

### Part 2

Our second goal was to investigate the extent to which providers met the new benchmarks for prevention strategies and whether oversight and guidance strategies employed by LME-MCOs suggested a shift to environmental strategies through analysis of expenditures.

#### Success at reaching the benchmark targets

**Fig. 2:**
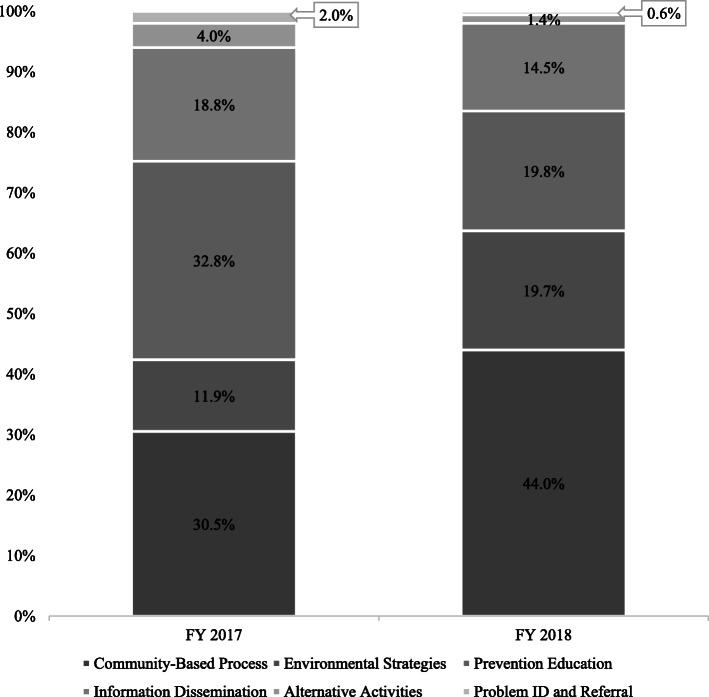
Percent of block grant Expenditures by CSAP Strategy and Year across NC Prevention System

In FY 2017, when the benchmarks were first introduced, LME-MCOs expended 42.4% of their block grant funds on community-based process and environmental strategies and 32.8% of their funds on prevention education (Fig. 2). However, in FY 2018, LME-MCOs expended 63.7% of their block grant funds on community-based process and environmental strategies and 20% of their funds on prevention education (Fig. 2). This was a substantial change in expenditures across LME-MCOs, suggesting that they had pivoted and were meeting the new benchmarks. In FY 2017, only one of the seven LME-MCOs had met the CORE strategy benchmarks. By FY 2018, all but one LME-MCO had met the CORE strategies benchmarks.

#### Success at reaching the benchmark targets across LME-MCOs and CSAP strategies

In addition, there was considerable variation by LME-MCO in their shift to meet the new benchmarks, both in comparison to other LME-MCOs and over time. To further explore the role of LME-MCO support and oversight, we examined benchmark implementation by LME-MCO oversight style. The graphs below show the shift to new benchmarks by LME-MCO according to their level of oversight from our coding into *intensive oversight* (LME-MCOs 1 and 2) and *facilitative oversight* (LME-MCOs 3–6). We found that the two high oversight LME-MCOs made the greatest progress in transitioning to the benchmarks.

##### Community-based processes and environmental strategies.

**Fig. 3:**
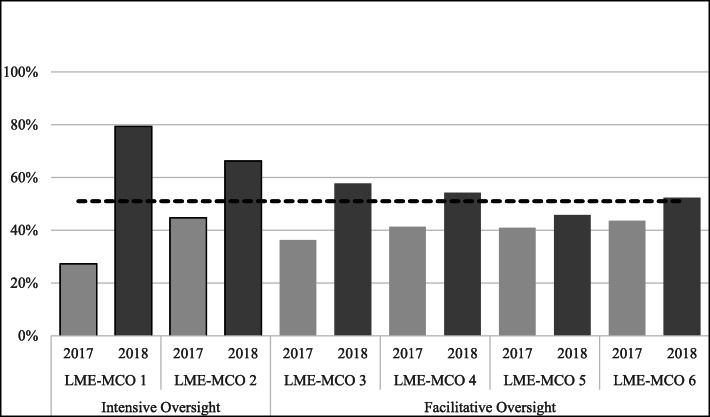
Percent of Expenditures on Community-Based Processes and Environmental Strategies by LME-MCO Agencies by year (2018 target benchmark = 51% or more spent in this category)

The new benchmarks set a target of LME-MCOs’ expending at least 51% of their allocated block grant funds on community-based processes and environmental strategies. LME-MCO 1 needed a greater degree of change to meet the new benchmark, whereas LME-MCO 2 needed relatively less change to meet new benchmarks on these two strategies. However, these two LME-MCOs (the intensive oversight group) reported the highest proportion of expenditures on community-based and environmental strategies in FY 2018 (Fig. 3).

##### Prevention education.

**Fig. 4:**
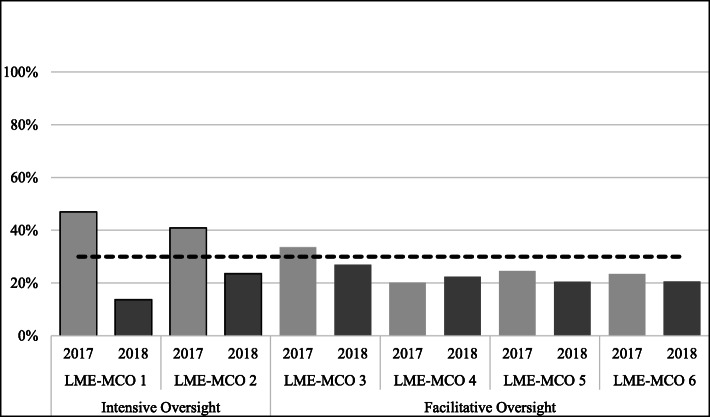
Percent of Expenditures on Prevention Education by LME-MCO Agencies by year (2018 target benchmark = 30% or less spent in this category)

The new benchmarks also set a target of LME-MCOs’ expending no more than 30% of their block grant funds on prevention education. All LME-MCOs met this benchmark in 2018 (Fig. 4); however, LME-MCO 1 and 2, the intensive oversight LME-MCOs, were farther from meeting this benchmark in FY 2017, and as such, had a greater degree of change to meet this benchmark.

##### Support strategies.

**Fig. 5:**
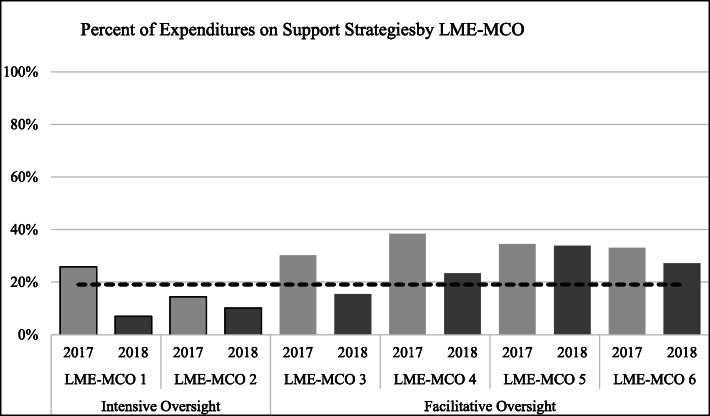
Percent of Expenditures on Support Strategies by LME-MCO Agencies by year (2018 target benchmark = 19% or more spent in this category)

The benchmarks set a target of LME-MCOs’ expending no more than 19% of their block grant funds on support strategies. Both LME-MCOs in the intensive oversight group met this benchmark in 2018, but only one of the four LME-MCOs in the *facilitative oversight* group did (Fig. 5).

## Discussion

This study examined how a complex state prevention system communicated and responded to a major organizational policy change, promoting a shift to environmental and community-based prevention strategies in North Carolina. We sought the perspectives of LME-MCO stakeholders as they are responsible for administering block grants in the NC prevention system. Overall, we found that participants (i.e., LME-MCO stakeholders) perceived the shift to new benchmarks positively and uncovered key aspects of the transition that made the shift achievable. We also documented specific changes they made to meet the new benchmarks and found that those with more intensive oversight strategies were more fully able to meet the benchmarks.

Several findings deserve highlighting. First, participants perceived a largely successful shift to the new benchmarks. Given the magnitude of the shift, it is notable that the participants we interviewed from the LME-MCOs shared this perception. Specifically, the shift appeared to be successful because of state guidelines that clearly articulated the new requirements and provided concrete guidance to assist LME-MCOs to make this change. This support came from both the state and the TTA center.

We also found that LME-MCOs made tangible progress toward meeting the benchmarks, particularly in the area of providing increased monitoring and oversight of the prevention portion of the block grant. Participants discussed a shift to providing more diverse methods of oversight to prevention providers. Importantly, some participants also noted that they felt constrained in changing the mix of providers in their network to better meet the benchmarks. Most participants believed that they had to work with the existing providers in their network and find ways to help providers shift to new strategies. Although it was beyond the scope of the present study, future work should examine the extent to which prevention providers, such as the existing providers mentioned by study participants, are equipped to deliver environmental prevention strategies. It is also important to note that given the way the block grant budget is allocated, LME-MCOs are not provided with funding to administer the block grants, thus potentially limiting staff time they are able to dedicate to this purpose.

Most LME-MCOs met the benchmarks earlier than anticipated—within 1 year. This was especially true for the LME-MCOs that provided a high level of oversight of their provider network. Although our data do not support causal inferences, the pattern of changes from funding allocations by CSAP strategies from FY 2017 to 2018 suggested that the two LME-MCOs who employed intensive oversight showed greater use of environmental and community-based strategies in 2018. Additionally, they had lower use of prevention education and support strategies, regardless of their 2017 starting points, compared to the LME-MCOs that employed less intensive oversight. This aligns with prior assessments of state prevention systems, which find that evaluation and monitoring are key in prevention systems [12, 13].

### Limitations and future directions

The contributions of this study should be interpreted in light of its limitations. First, both of our sources of data (LME-MCO interviews and expenditure data) relied on self-reports. As with all qualitative research, participants might have been motivated to present their activities and opinions positively. We made efforts to minimize this by emphasizing that all information would be kept confidential. Relatedly, we cannot ascertain from the present data the *quality* of LME-MCO monitoring and oversight of providers. Future research can help elucidate the nature and quality of oversight and guidance given to providers and the nature and quality of the prevention programming being utilized by providers. The increase in environmental and community-based processes shown in the CSAP strategy data might be artificially inflated in FY2018 given that providers completed a needs assessment in FY2018, which was coded as time/money spent on community process.

Importantly, while this study examined whether the state prevention system was able to shift to new benchmarks successfully, we could not evaluate whether the shift predicted substance misuse outcomes among the populations the LME-MCO and prevention providers serve. There is some indication that NC DMH’s directive regarding environmental strategies coincided with improvements in three substance misuse outcomes (alcohol-involved fatal crash rate, acute alcohol-attributable death rate, and numbers of Emergency Department visits involving heroin [44]. It is important for future studies to further investigate how LME-MCOs and individual providers, determine which prevention strategies to implement. Finally, it is not clear how generalizable these findings are given the variability in state prevention system implementation [13]; however, the findings still contribute to understanding policy shifts in complex prevention systems.

## Implications and conclusion

This study contributes to the prevention literature in several ways. First, it provides an account of a major state-level shift in prevention policy from the perspective of regional entities directly involved in managing the shift. Second, the study identifies specific practices that appear to have been helpful in implementing a major shift in prevention policy (e.g., the need for clear communication and guidance about the policy changes and active monitoring and oversight by LME-MCOs). Third, LME-MCOs varied in their provision of oversight and guidance to prevention providers; higher oversight and guidance—such as using an RFP to bid out the system of providers, using a model scope of work, and using an auditing or formal review system – appeared to be associated with providers meeting the new benchmarks. Finally, participants revealed a sense that the managed care system with regional entities administering prevention funding is unnecessarily complex and may leave regional entities feeling constrained in their roles. For system change efforts to succeed in complex systems, such as North Carolina’s state prevention system, implementing agencies need a high level of support and communication about policies they are to implement and need to develop strong oversight and guidance strategies to support organizations who deliver programming.

## Data Availability

The datasets generated during and/or analyzed during the current study are not publicly available due to the nature of the interview data (which might be identifiable given the small number of organizations represented in our sample) but are available from the corresponding author on reasonable request.

## Abbreviations

SABG: Substance Abuse Prevention and Treatment Block Grant
SAMHSA: Substance Abuse and Mental Health Services
NC DHHS: North Carolina Department of Health and Human Services
TTA Center: Training and Technical Assistance Center
SSA: Single State Agency
LME-MCO: Local Management Entities/Managed Care Organizations
NC DMH: North Carolina Department of Mental Health, Developmental Disabilities, and Substance Abuse Services
CSAP: Center for Substance Abuse Prevention
SPF: Strategic Prevention Framework
NC: North Carolina
